# Impact of the COVID-19 pandemic on the mental health and well-being of Veterans’ spouses: a cross sectional analysis

**DOI:** 10.1186/s12888-023-04687-y

**Published:** 2023-03-22

**Authors:** Kevin T. Hansen, Rachel A. Plouffe, Deanna L. Walker, Sonya G. Wanklyn, Laryssa Lamrock, Polliann Maher, Anthony Nazarov, J. Don Richardson

**Affiliations:** 1 MacDonald Franklin OSI Research Centre, Lawson Health Research Institute, London, ON Canada; 2 Department of Psychiatry, Western University, London, ON Canada; 3 Department of Psychology, Western University, London, ON Canada; 4 St. Joseph’s OSI Clinic, Parkwood Institute, St. Joseph’s Health Care London, London, ON Canada; 5 The Atlas Institute for Veterans and Families, Ottawa, ON Canada; 6 Department of Psychiatry and Behavioural Neurosciences, McMaster University, Hamilton, ON Canada

**Keywords:** Military spouses, Mental health, Well-being, Telehealth use, COVID-19

## Abstract

**Background:**

COVID-19 has negatively impacted the mental health and well-being of both Canadians and the world as a whole, with Veterans, in particular, showing increased rates of depression, anxiety, and PTSD. Spouses and common-law partners often serve as primary caregivers and sources of support for Veterans, which may have a deleterious effect on mental health and increase risk of burnout. Pandemic related stressors may increase burden and further exacerbate distress; yet the effect of the pandemic on the mental health and well-being of Veterans’ spouses is currently unknown. This study explores the self-reported mental health and well-being of a group of spouses of Canadian Armed Forces Veterans and their adoption of new ways to access healthcare remotely (telehealth), using baseline data from an ongoing longitudinal survey.

**Methods:**

Between July 2020 and February 2021, 365 spouses of Veterans completed an online survey regarding their general mental health, lifestyle changes, and experiences relating to the COVID-19 pandemic. Also completed were questions relating to their use of and satisfaction with health-care treatment services during the pandemic.

**Results:**

Reported rates of probable major depressive disorder (MDD), generalized anxiety disorder (GAD), alcohol use disorder (AUD), and PTSD were higher than the general public, with 50–61% believing their symptoms either directly related to or were made worse by the pandemic. Those reporting being exposed to COVID-19 were found to have significantly higher absolute scores on mental health measures than those reporting no exposure. Over 56% reported using telehealth during the pandemic, with over 70% stating they would continue its use post-pandemic.

**Conclusions:**

This is the first Canadian study to examine the impact of the COVID-19 pandemic specifically on the mental health and well-being of Veterans’ spouses. Subjectively, the pandemic negatively affected the mental health of this group, however, the pre-pandemic rate for mental health issues in this population is unknown. These results have important implications pertaining to future avenues of research and clinical/programme development post-pandemic, particularly relating to the potential need for increased support for spouses of Veterans, both as individuals and in their role as supports for Veterans.

## Background

Research has highlighted the negative impact of the COVID-19 pandemic on the general health and well-being of Canadians [1] and globally [1–3]. This is especially pertinent for at-risk populations such as Veterans [4, 5], given that Veterans experience psychiatric conditions including posttraumatic stress disorder (PTSD), major depressive disorder (MDD), generalized anxiety disorder (GAD) and alcohol use disorder (AUD) at higher rates compared to age- and sex-matched Canadian civilians [6–9]. Studies investigating how the COVID-19 pandemic has affected the mental health of Veterans have been mixed, with some studies finding there to be no effect [10], while others found a worsening of mental health severity [11, 12]. Social distancing in particular has been associated with an increased severity of symptoms in Veterans with pre-existing mental health conditions [13].

While not every Veteran experiences mental or physical difficulties as a result of their service, for those who do, their spouses often serve as caretakers and the main support for both the Veteran and their families, which can subsequently lead to increased stress and risk of burnout for the spouses [14, 15]. To date, research evaluating the mental health of Veterans’ spouses has predominantly focused on how the spouse’s health is affected by their active-duty service member being away on deployment/stationed away from their families, or by the presence of physical and mental health challenges in the Veteran [16]. Thus, research has been conducted through a Veteran-focussed lens. Few studies have explicitly investigated the mental health of spouses as a topic of interest in and of itself despite the importance of spouses to the Veteran’s physical and mental well-being implied by the aforementioned research, and none have looked at the effect of the COVID-19 pandemic on this group.

Lifestyle changes imposed by the pandemic and its associated restrictions have served to increase the amount of contact between spouses, and their Veteran partners, while decreasing the availability of respite opportunities outside the home. Public safety guidelines, for example, have exacerbated work instability and financial difficulties reported by veterans and their families [17], decreased their access to many important services and professional supports, and have significantly reduced individuals’ abilities to engage in social interactions that previously provided an outlet for dealing with increased stress. The latter is of particular concern for spouses and their Veteran partners who may already experience limitations regarding social engagement [18, 19]. These additional pandemic stressors may serve to exacerbate individuals’ existing mental health conditions, increasing the need for accessible mental health support and services [13, 20, 21]. It is plausible, therefore, that the lifestyle and occupational restrictions introduced by the COVID-19 pandemic, in particular decreased access to health care, peers, and social support, may further exacerbate the challenges that spouses may already be experiencing with regards to both their Veteran partner and their own mental health and well-being.

As not much research has been done that looks specifically at the mental health and well-being of spouses of Veterans, the overarching aim of the current study is to explore the self-reported mental health and well-being of the spouses of Canadian Armed Forces (CAF) Veterans, as well as their willingness to adopt new ways of accessing required healthcare services (i.e., via telehealth) during the COVID-19 pandemic. These results will serve to provide a starting point for further research to increase understanding of the general mental health and well-being of Veterans’ spouses, as well as information around how these areas are affected by the increased strains imposed by global events such as pandemics.

## Methods

A national longitudinal online survey was distributed across Canada to English- and French-speaking CAF Veterans and spouses/partners of Veterans (see Forchuk et al. [22] for specifics around study design). Participants were recruited using professional networks, social media advertisements, participant recruitment websites, press releases, Veteran community and advocacy groups, and word of mouth. The survey was open to all CAF Veterans and spouses/partners of Veterans over the age of 18 residing in Canada during the baseline data collection period (July 7, 2020 to February 1, 2021). The survey was hosted online via the Research Electronic Data Capture (REDCap) platform, with participants being invited to complete either a short form (20 min) or long form (30 min) survey. Participants were not offered remuneration for study participation. The spousal baseline data drawn from this large longitudinal study [22] serves as the data source for the current study. Results from a previous study of Veterans using identical sampling methods [12] found that 77% of Veterans reported being married/in a relationship. Using this figure as an expected prevalence rate, a 95% CI, and an alpha level of 0.05, the required sample size was determined to be 275 individuals.

### Measures

#### COVID-19-related factors

Personal and familial exposure to, and consequences of COVID-19, since the beginning of the pandemic, were assessed using items drawn from the Coronavirus Health and Impact Survey (CRISIS [23]). Participant responses to items indicative of COVID-19 exposure were used to create a dichotomous “COVID-19 exposure” variable (exposed vs. not-exposed). Endorsement of any of the following questions was considered a positive COVID-19 exposure: (1) exposure to someone with suspected or confirmed COVID-19, (2) having a family member with suspected or confirmed COVID-19, (3) personal suspected or confirmed COVID-19, (4) experiencing personal consequences resulting from COVID-19 (e.g., illness, quarantine, hospitalization), or (5) familial consequences resulting from exposure to COVID-19. Individuals who did not endorse any of the previous questions were considered to have a negative COVID exposure for this study. Additional items were used to assess pandemic-related changes in employment status or setting, income, and concerns about employment stability.

### Depression symptoms

The Patient Health Questionnaire (PHQ-9; [24, 25]) was used to assess for probable MDD. Participants rated how frequently they experienced each of nine symptoms in the preceding 2 weeks on a 4-point scale ranging from 0 (*not at all*) to 3 (*nearly every day*). A score of 10 or higher is indicative of probable depression, while suicidal ideation is indicated by any response other than 0 (*not at all*) on the associated PHQ-9 question (i.e., “*Thoughts that you would be better off dead or of hurting yourself in some way*”). Internal consistency of the current sample was high (Cronbach’s α = 0.91).

### General anxiety symptoms

The General Anxiety Disorders-7 (GAD-7 [26, 27]) was used to assess for probable GAD. Participants were asked how bothered they had been by seven anxiety symptoms over the past two weeks with responses ranging from 0 (*not at all*) to 3 (*nearly every day*). A score of 10 or more is indicative of probable generalized anxiety disorder [27]. Internal consistency of the current sample was high (Cronbach’s α = 0.94).

### Alcohol use

The Alcohol Use Disorders Identification Test (AUDIT; [28]) was used to assess for past year probable AUD. The AUDIT is a 10-item, self-report measure, with response options of 0 (*never*) to 4 (*4 or more times a week*) for drinking frequency questions; 0 (*none*) to 4 (*10 or more*) for drinking quantity questions; 0 (*never*) to 4 (*daily or almost daily*) for questions around drinking consequences; and 0 (*no*), 2 (*yes, but not in the past year*), or 4 (*yes, during the past year*) for concern of others about participant’s drinking and experiencing or causing injury as a result of their drinking. Potential scores ranged from 0 to 40, with scores of 7 (for women) and 8 (for men) or above indicating probable AUD [28]. Internal consistency of the current sample was good (Cronbach’s α = 0.82).

### Posttraumatic stress disorder (PTSD) symptoms

The PTSD Checklist for the DSM-5 (PCL-5; [29]) assessed past month probable PTSD and PTSD symptom severity based on DSM-5 symptom clusters. Respondents rated their distress from each of the 20 items from 0 (*not at all*) to 4 (*extremely*). Responses were summed to provide a total score, where higher summed scores indicated greater PTSD symptom severity. A score of 33 or higher is indicative of probable PTSD [30–32]. Internal consistency of the current sample was high (Cronbach’s α = 0.97).

### Self-perceived mental health

To determine if the reported mental health symptoms were related to the pandemic, an extra item was added to the end of PHQ-9, GAD-7, and PCL-5. This additional item asked if the reported symptoms were directly related to, made worse by, or were unrelated to the pandemic. Additionally, spouses were asked to rate their overall current mental health in response to the following question: ‘*Compared to before the start of the COVID-19 pandemic, would you say your mental health is …’ *using a 5-point scale ranging from 1 (*significantly worse than before*) to 5 (*significantly better than before*). The same question and response options were then provided regarding their perceived current stress level compared to before the pandemic. Finally, participants were also asked to rate their Veteran partner’s respective mental health and stress levels using the same ratings on similar questions (i.e., ‘Compared to before the start of the COVID-19 pandemic, would you say your spouses’ mental health [stress level] is …’ using a 5-point scale ranging from 1 (significantly worse than before) to 5 (significantly better than before).

### Access to healthcare and telehealth use

Participants were asked about difficulties accessing health care they experienced, whether these access difficulties related to mental or physical healthcare, and how much distress these difficulties had caused them in the previous week using a 5-point scale ranging from 0 (*not at all*) to 4 (*extremely*). If individuals indicated that they had utilized telehealth services, satisfaction with these telehealth services (e.g., ‘*I would recommend telehealth to a friend’* and ‘*I would choose to use telehealth in the future if coming to the office was inconvenient’*) were assessed on a scale ranging from 1 (*strongly disagree*) to 4 (*strongly agree*).

### Data analytic strategy

For the current sample, a missing data cut-off was established at 20% or less, and treatment of missing data was addressed via pairwise deletion [33, 34]. An alpha level of 0.05 was used for statistical significance in all tests. Analyses were conducted using IBM SPSS Statistics, Version 23 [35]. Sociodemographic characteristics, employment characteristics, mental health functioning, and mental health symptoms were assessed using descriptive statistics. Percentage of participants who would recommend telehealth to a friend or use telehealth in the future themselves were determined by combining ‘*agree*’ and ‘*strongly agree*’ responses. The overall perceived change in mental health and stress levels due to the COVID-19 pandemic was calculated by combining *“slightly and significantly worse”* and “*slightly and significantly better”* responses. Associations of spouses mental health symptoms (via PHQ-9, GAD-7, AUDIT, and PCL-5 scores) with COVID-19 exposure (exposed vs. not exposed), and with Veteran partners’ diagnosis with either a mental health condition or a mental health condition related to their occupation, were explored using independent samples *t*-tests. Associations between mental health symptoms and age were explored using bivariate Pearson correlations, while mental health symptoms and education and income were explored using bivariate Spearman correlations. Chi square analyses were used to examine the association between age and telehealth variables with strength of associations interpreted using eta value, with eta squared used to determine amount of variance accounted for.

## Results

### Sample characteristics

Baseline assessment surveys were completed by 365 individuals who identified as being spouses or partners of a Canadian Veteran (see Table 1). The mean age was 49.9 years (*SD* = 13.56, range = 21–94 years). The sample was predominantly women (98.6%, *n* = 354), precluding any analysis by gender, and over 86% reported living with their spouse/common-law partner. The most common self-identified ethnicity was White (93.4%, *n =* 341) with Indigenous and Other (non-white) groups composing 4.1% and 4.7% of the sample, respectively. Geographically, the majority of respondents reported living in Central Canada (i.e., Ontario or Quebec; 43.9%, *n* = 148), with the overall pattern of distribution mirroring that of Veterans in Canada in 2021 [36], with most reporting living in small towns, villages, or in rural areas (48.5%, *n* = 175) as opposed to in and around large cities (27.7%, *n* = 100).

**Table 1:** Sociodemographic characteristics of Spouses of Veterans at baseline data collection

Variable	*n* or *M*	% or *SD*
Age (years)	49.99	13.56
Gender		
Men	5	1.40%
Women	354	98.60%
* Missing*	*6*	*1.6*
Marital Status		
Married/common-law	315	88.50%
Divorced/Separated/Widowed	35	9.80%
Single in long-term relationship	6	1.70%
* Missing*	*9*	*2.50%*
Ethnicity		
White	341	93.40%
Indigenous	15	4.10%
Other (non-white)	17	4.70%
* Prefer not to say*	*4*	*1.10%*
Education (highest level completed)		
Less than high school	14	3.90%
High school diploma	48	13.30%
Some college/university	93	25.80%
Completed college/university	205	56.90%
* Missing*	*5*	*1.40%*
Annual income (CND $)		
< 40,000	28	9.30%
40,000 to 59,999	32	10.60%
60,000 to 79,999	54	17.90%
80,000 to 99,999	54	17.90%
100,000 to 119,999	58	19.20%
> 120,000	76	25.20%
* Missing*	*63*	*17.30%*
Region		
Atlantic Provinces (NB, NL, NS, PE)	77	22.80%
Central Canada (ON, QC)	148	43.90%
Prairie Provinces (AB, MB, SK)	76	22.60%
West & Northern Territories (BC, NT, NU, YT)	36	10.70%
* Missing*	*5*	*1.40%*
Area of Residence		
Large city	49	13.60%
Suburb of large city	51	14.10%
Small city	86	23.80%
Town or village	100	27.70%
Rural area	75	20.80%
* Missing*	*5*	*1.40%*
Living Arrangements		
Live alone	35	9.60%
Live with spouse/common-law	318	86.80%
Live with children	178	48.80%
Live with parents/in-laws	14	3.80%
Live with other family	11	3.00%
Live with friends, roommates/other	11	3.00%

Note: Totals may exceed 100% as categories are not mutually exclusive

### Employment

The majority of Veterans’ spouses (61.3%, *n* = 224) reported being employed prior to the COVID-19 pandemic (see Table 2), with 26.5% (*n* = 95) reporting that their employment status changed in some way since the pandemic began. Depending on pre-COVID occupation status, the most commonly reported changes for those who were employed pre-COVID was becoming unemployed (20.5–47.8%). Changes in duties and/or responsibilities at work was the next most frequently occurring change being reported by between 7.3% and 22.0% of those previously employed. When directly asked how concerned they were about losing their job, 50.3% (*n* = 86) of those currently employed indicated that they were at least a little concerned.

**Table 2:** Proportion of participants reporting experiencing change in employment status during COVID-19 (*n* = 365)

		Became (or experienced) … during COVID					
		Unemployed	Employed	Change in duties or responsibilities	Furloughed or on paid leave	Change in benefits received	Other (none of the above)
Employment Status pre-COVID	*n*	%	%	%	%	%	%
Employed							
Full-time	151	20.50%	3.30%	7.30%	1.30%	4.00%	3.30%
Part-time	50	26.00%	4.00%	22.00%	6.00%	6.00%	6.00%
Self-Employed	23	47.80%	9.10%	11.10%	0%	4.30%	8.70%
Unemployed							
Looking for work	9	0%	22.20%	0%	0%	0%	0%
Due to illness	25	0%	0%	4.00%	4.00%	4.00%	0%
Other							
Student	12	8.30%	8.30%	8.30%	0%	16.70%	0%
Retired	75	1.30%	1.30%	0%	0%	0%	1.30%
Homemaker	33	3.00%	0%	3.00%	0%	3.00%	0%
Other	11	0%	0%	0%	0%	0%	3.60%

Note: Column totals may exceed total n or 100% as categories are not mutually exclusive

### Mental Health Functioning

The majority of spouses (70.1%, *n* = 232) felt their mental health had worsened since the beginning of the COVID-19 pandemic, and 83.3% (*n* = 269) felt that they were more stressed than they had been pre-COVID. This perceived worsening in mental health was significantly related to the spouses’ perceptions of their Veteran partner’s current mental health (*r*[305] = 0.41, *p* < .001) and stress levels (*r*[305] = 0.38, *p* < .001) relative to pre-COVID with 70.2% and 75.7%, respectively, believing their partners were currently worse than before COVID. The perceived worsening of spouses’ stress levels was also significantly related to their perceptions of their Veteran partner’s current mental health (*r*[302] = 0.38, *p* < .001) and stress levels (*r*[302] = 0.55, *p* < .001) relative to pre-COVID. Having a Veteran partner who was formally diagnosed with a mental health condition was also associated with the spouses’ perceived mental health (χ^2^[1, *n* = 313] = 7.17, *p* = .03), but not spouses’ perceived stress levels (χ^2^[1, *n* = 308] = 2.70, *p* = .26).

Having a Veteran partner whose mental health condition was related to their occupation was found to be associated with the spouses’ perceived mental health (χ^2^[1, *n* = 303] = 6.98, *p* = .03) but not stress levels (χ^2^[1, *n* = 298] = 5.65, *p* = .06). When the spouses’ current mental health symptoms (measured by the PHQ-9, GAD-7, AUDIT, and PCL-5) were compared according to their Veteran partner’s status regarding being diagnosed with a mental health condition or if their mental health condition was related to their occupation, no difference was found between the two groups on any measure with the exception of symptoms of depression (*t*[191.6] = 2.24, *p* = .027) and PTSD (*t*[183.0] = 2.22, *p* = .028) for those whose partner had a mental health condition related to their occupation (see Table 3).

**Table 3:** Veteran spouse mental health measure scores by Veteran mental health diagnosis statuses

		Is your spouse currently diagnosed with a mental health condition?			
		Yes	No		
Measure	*df*	*M (SD)*	*M (SD)*	*t-statistic*	*p-value*
PHQ-9	219	11.11 (7.02)	9.50 (6.53)	1.72	0.087
GAD-7	219	9.11 (6.29)	8.21 (5.64)	1.09	0.278
AUDIT	193	3.61 (4.77)	2.65 (3.03)	1.57	0.118
PCL-5	217	29.30 (22.66)	25.67 (20.52)	1.20	0.232
		**Is your spouse’s mental health diagnosis related to their occupational service?**			
		**Yes**	**No**		
**Measure**	***df***	***M (SD)***	***M (SD)***	***t-statistic***	***p-value***
PHQ-9	191.6	11.31 (7.15)	9.30 (5.87)	2.24	0.027
GAD-7	192.4	9.33 (6.33)	8.06 (5.27)	1.58	0.115
AUDIT	186.2	3.64 (4.74)	2.75 (3.11)	1.57	0.119
PCL-5	183.0	30.16 (22.67)	23.63 (19.56)	2.22	0.028

Note: AUDIT = Alcohol Use Disorders Identification Test, GAD-7 = Generalized Anxiety Disorde-7, PCL-5 = Post-traumatic Stress Disorders Checklist, PHQ-9 = Patient Health Questionnaire

The proportion of Veterans’ spouses who met screening criteria for probable mental disorders ranged from 12.9 to 49.1% on measures of alcohol use disorders (12.9%, *n* = 26), GAD (38.0%, *n* = 89), PTSD (38.9%, n = 89), and MDD (49.1%, *n* = 115), with 21.8% (*n =* 51) reporting significant suicidal ideation. When asked if they felt their current mental health symptoms were related to the COVID-19 pandemic, 61.0% (*n* = 136), 66.4% (*n* = 146), 50.5% (*n* = 104) of spouses said their depression, anxiety, and PTSD symptoms, respectively, were either directly related to or made worse by the pandemic.

To evaluate differences in mental health outcomes, *t*-tests were used to compare group means on MDD, GAD, AUD, and PTSD severity by COVID-19 exposure, age, educational attainment and income. Distribution of data was examined and both skew and kurtosis were found to fall within acceptable ranges for normality using cut offs established by Kline [37] of ± 3 and 10, respectively. Results showed that exposure to COVID-19 was associated with higher scores on measures of depression, anxiety, and PTSD severity but not in terms of AUD severity (*p* = .58) (see Table 4). Similarly, younger age was also associated with increased depression (*r*[218] = − 0.33, *p* < .001), anxiety (*r*[218] = − 0.42, *p* < .001), and PTSD severity (*r*[213] = − 0.29, *p* < .001). In contrast, educational attainment and income were found to be statistically associated only with AUD severity (*r*[203] = 0.15, *p* = .33; *r*[174] = 0.26, *p* = .001), respectively.

**Table 4:** Mental health measure scores by COVID-19 exposure status

		COVID exposed	Not COVID Exposed		
Measure	*df*	*M (SD)*	*M (SD)*	*t-statistic*	*p-value*
PHQ-9	220	11.72 (6.79)	8.29 (6.26)	3.91	< 0.001
GAD-7	220	9.17 (5.96)	7.34 (5.91)	2.30	0.022
AUDIT	193	3.11 (4.05)	2.80 (3.23)	0.55	0.583
PCL-5	215	30.04 (21.95)	21.72 (19.66)	2.92	0.004

Note: AUDIT = Alcohol Use Disorders Identification Test, GAD-7 = Generalized Anxiety Disorde-7, PCL-5 = Post-traumatic Stress Disorders Checklist, PHQ-9 = Patient Health Questionnaire

Of the spouses who responded, over half (56.1%, *n* = 170) reported experiencing difficulty accessing healthcare, with the majority reporting difficulties accessing both physical and mental health care (53.3%, *n* = 90), followed by physical health care (40.2%, *n* = 68), and mental health care (6.5%, *n* = 11). Access to primary care, dental, and specialist care services were the most frequently reported access challenges. While mental health care access had the lowest reported rate of access difficulty, 64% (*n* = 64) of respondents indicated that it caused them moderate to extreme distress, only slightly behind physical care access (67.5%, *n* = 106).

At time of data collection, 41.4% (*n* = 126) of spouses reported receiving health care via telehealth services with the majority reporting this care related to physical care (27.8%, *n* = 98). When asked if they would continue to use telehealth if face-to-face physical care remained inconvenient, 70.5% (*n* = 67) indicated that they would, with 74.2% (*n* = 69) stating they would recommend it to a friend. Those who reported using telehealth for mental health care (17.3%, *n* = 61), 71.2% (*n* = 42) of respondents indicated that they would continue to use the service if face-to-face were inconvenient, with 72.9% (*n* = 43) indicating they would recommend it to a friend. Age was found to have a significant association with both continuing to use telehealth services for physical (η = 0.74, η^2^ = 0.55) and mental (η = 0.81, η^2^ = 0.66) health care, and recommending telehealth to their friends for physical (η = 0.71, η^2^ = 0.50) and mental (η = 0.81, η^2^ = 0.65) health care.

## Discussion

Our findings provide an important first-look at the mental health and well-being of Veterans’ spouses and how these aspects of their lives has been impacted by the COVID-19 pandemic. Our results showed that 70% of spouses believed that COVID-19 had a negative effect on their overall mental health. This is almost twice the rate reported by the Canadian Mental Health Association in their survey of a representative sample of 3,000 Canadians asking a similar question (38%) [38]. Self-reported symptoms of specific mental health conditions were also significantly higher than those found in the general public post-COVID-19 onset. The reported rates of probable major depressive disorder (MDD) in Veterans’ spouses were found to be more than double that of the general population (49% vs. 19%), as were the rates for probable generalized anxiety disorder (GAD) (38% vs. 15%), and rates for probable PTSD were over five-times the rates seen in the general population (39% vs. 7%) [39]. Interestingly, the rates found among spouses were also higher than those reported by CAF Veterans for the same conditions (MDD = 35%, GAD = 27%, PTSD = 34%), though the rates for alcohol use disorder (AUD) did not differ between Veterans and Veterans’ spouses (13%) [12]. These findings, in part, may be attributable to the negative impact of social isolation on the already-vulnerable population, specifically being cut off from their usual avenues of formal and informal support. Having been personally exposed to COVID-19 was also found to be associated with higher scores on measures of probable mental health conditions suggesting that the mental health of spouses of Veterans was particularly affected by the pandemic, an interpretation reinforced by over half of the respondents reporting that COVID-19 had made their depression, anxiety, and PTSD symptoms worse.

While other researchers have also found similar patterns of higher rates of mental health issues in Veterans’ spouses than the general public, these findings are somewhat inconsistent in terms of their abilities to reach statistical significance (see Armour [16] for review). In addition, most of these studies were conducted with spouses of Veterans who themselves were experiencing mental health issues (e.g., PTSD), which may have confounded the interpretation of those spousal scores. Unfortunately, since no research has been done to-date that looks at the mental health of spouses of Veterans without co-morbid mental health issues, the exact effect of the increased stress applied by COVID-19 cannot be determined. It is notable that much of the prior research has been conducted by agencies and institutions whose mandates are focussed on assisting Veterans’ experiencing mental and/or physical challenges associated with their prior service (e.g., Veteran’s Affairs Canada, Department of Veteran’s Affairs [USA], Office of Veterans’ Affairs [UK]). Further study specifically into the mental health of the spouses of “well” Veterans is required to establish a baseline with regards to spousal rates of various mental health conditions.

The use of a telehealth format for healthcare service delivery was found to be generally well accepted by participants despite the somewhat abrupt and involuntary nature of its adoption. The majority of spouses who used telehealth services during the early part of the pandemic viewed it favourably with most indicating they were open to continuing to access both physical and mental health services this way, even post-pandemic. Being able to access healthcare from home, especially mental healthcare, could potentially make seeking out and continuing treatment more appealing for those who do not live close to large cities where services are often provided, and those for whom travelling may be difficult. Almost two-thirds of respondents in this study stated that an inability to access mental health related services in particular caused them significant distress, indicating that telehealth may be an important area for future clinical, programme, and infrastructure development focus for Veterans’ spouses.

Although it is evident that rates of mental health conditions have been exacerbated by the COVID-19 pandemic, many Veterans are reticent to seek out treatment for a variety of reasons, including a persistent stigma around mental illness [40–42]. Consequently, those who have sought treatment and have received diagnoses are likely to be those who have/are experiencing more severe symptoms. When a sample of Canadian Veterans was asked to compare their current level of mental health functioning to their level prior to the pandemic, 56% of respondents indicated that it had become worse [12]. This makes the finding that 70% of the responding spouses believed their Veteran partner’s mental health had worsened compared to pre-pandemic, an area for further study. The difference in responses could reflect either that the Veterans spouses may be better able to detect subtle changes in their partners’ functioning than the partners can themselves, or alternately, spouses may be more willing to report a perceived worsening in their partners functioning (i.e., the Veterans may be less likely to report worsening due to the aforementioned stigma relating to mental health conditions). The ability for Veterans’ spouses to detect subtle changes in their partners’ mental health states due to their near daily contact with the Veteran may provide another avenue for symptom monitoring that would benefit from future study.

The results of this study should be viewed in light of the following limitations. The current study made use of data gathered early in the course of the pandemic (July 2020 to February 2021). As the pandemic developed and restrictions changed, the effect of the pandemic on mental health of individuals may have subsequently changed. As more longitudinal data become available, future studies should evaluate the effect that the various waves and increasing rates of infection and death had on individual functioning and overall well-being.

Second, as participation in the study was based on self-selection, it is possible that spouses who experienced more mental health issues themselves were more likely to complete the survey. Similarly, those spouses whose Veteran partners have mental health issues may also have self-selected for a similar reason. Third, study participants were recruited using a convenience-snowball sampling methodology which may limit the generalizability of results to the larger Veteran spouse community. Based on these preliminary results, however, a larger study using a representative sample of this population would allow for a clearer picture of the mental health and well-being of Veterans’ spouses to be developed. Fourth, as the survey was completed electronically, this required that potential participants have access to a web-enabled device (e.g., smart phone, tablet, computer) and sufficient familiarity with the technology to use it effectively. Therefore Veterans’ spouses who do not have ready access to the internet, a computer, or smart phone/devices, or who are less familiar with the use of digital devices may be underrepresented in our sample [43]. This may have affected sampling in two ways: the sample may primarily represent those with more financial means (i.e., they must be able to afford these devices), and participants’ ages may be on average younger, as older individuals may feel less comfortable using technology and completing surveys online. However, web surveys are becoming more commonplace and technology more ubiquitous, so it is plausible that this did not impact our findings in a significant way. Finally, as the data gathered were cross-sectional in nature, this limits the type of conclusions that can be drawn. Thus, the use of longitudinal data would allow more diverse and potentially informative conclusions with regards to causal relationships between variables.

## Conclusions

To our knowledge, this is the first Canadian study examining the impact of the COVID-19 pandemic on spouses of Veterans. Our study found that Veterans’ spouses reported worsening mental health and/or symptom exacerbation as a result of the COVID-19 pandemic, though the base rates of existing mental health issues in this population still remains unknown and needs further research. Our results have important implications for both clinicians and program administrators. It highlights the need for more supports being available specifically for the spouses of Canadian Veterans to help with the various stresses they encounter, both with respect to the spouses’ mental health itself and also given the potential for indirect benefit to their Veteran partners.

## Data Availability

The datasets from the current study are not publicly available due to being part of a longitudinal research project. Data is available from the corresponding author on reasonable request.

## List of abbreviations

AUD: Alcohol use disorder
AUDIT: Alcohol Use Disorders Identification Test
CAF: Canadian Armed Forces
CRISIS: Coronavirus Health and Impact Survey
df: degrees of freedom
GAD: Generalized anxiety disorder
GAD-7: Generalized Anxiety Disorders-7
M: Mean
MDD: Major depressive disorder
OSI: Operational Stress Injury
PCL-5: PTSD Checklist for the DSM-5
PHQ-9: Patient Health Questionnaire
PTSD: Posttraumatic stress disorder
SD: Standard deviation
